# Enhancing Surgical Safety and Efficiency: Systematic Review and Single-Arm Meta-Analysis of Surgical Data Recorders

**DOI:** 10.2196/72703

**Published:** 2025-07-28

**Authors:** Niels Siegel, André Rotärmel, Georgios Polychronidis, Gabriel Salg, Rosa Klotz, Pascal Probst, Thomas Pausch

**Affiliations:** 1 Department of General, Visceral and Transplantation Surgery, University Hospital Heidelberg, Im Neuenheimer Feld 420, Heidelberg, 69120, Germany, 49 6221-56-6110; 2 University Center for Orthopedics, Trauma and Plastic Surgery, Carl Gustav Carus Hospital, Dresden, Germany; 3 Harvard TH Chan School of Public Health, Harvard University, Cambridge, MA, United States; 4 Department of Surgery, Kantonsspital Frauenfeld, Frauenfeld, Switzerland; 5 Partnership Between DKFZ and University Medical Center Heidelberg, National Center for Tumor Diseases, Heidelberg, Germany

**Keywords:** patient safety, operating room, safety behavior, outcome assessment, surgical data recorders, surgical performance, intraoperative monitoring, meta-analysis, healthcare technology, medical error reduction

## Abstract

**Background:**

Recently, surgical data recorders that are comparable to flight data recorders, also known as black boxes in the aviation industry, have been developed to improve patient safety and performance in surgery. These devices allow for unique insights in the operating room by providing new data capture capabilities. No systematic review has been carried out to evaluate the areas of application of surgical data recorders to date.

**Objective:**

This systematic review and single-arm meta-analysis aims to assess the aspects of the operating theater environment for which surgical data recorders are used and to make a preliminary assessment of the quantifiable data that can be collected, compared to traditional collection methods.

**Methods:**

This systematic review followed the PRISMA (Preferred Reporting Items for Systematic Reviews and Meta-Analyses) guidelines. Medline, Embase, and Web of Science databases were lastly systematically searched for papers that focused on a clinical use case for surgical data recorders on February 10, 2025. In particular, not relevant papers focusing on implementation of surgical data recorders were excluded. Title, abstract, and full-text screening were completed to identify relevant articles. The included studies were analyzed descriptively using data extraction forms. Where possible, quantifiable data was also analyzed. Risk of bias was assessed using the Risk Of Bias In Non-Randomized Studies of Exposure (ROBINS-E) tool.

**Results:**

In total, 70 studies were screened, and a total of 17 studies were included. A total of 10 of the 17 studies had a low overall risk of bias; however, confounding, selection bias, small sample sizes, short study periods, and potential Hawthorne effects were the notable limitations. Only 2 studies were assessed to have publication bias. Use cases could be grouped into 4 categories: economic, safety, behavior in the operating room, and technical skill assessment. A single-arm meta-analysis focusing on adverse events and distractions in the operating theater could be conducted, demonstrating accurate reporting of distractions in line with the existing literature.

**Conclusions:**

Surgical data recorders provide an unobstructed view of various aspects of the operating theatre. Most published papers present preliminary studies on surgical data recorders, indicating the potential for further, larger-scale studies with enhanced methodological quality.

## Introduction

Since the 1960s, aviation has relied on flight recorders—commonly known as “black boxes”—to capture flight data for postincident analysis, enabling investigators to understand failures and drive safety improvements [1,2]. The operating room (OR), much like the cockpit, is a high-stakes, high-complexity environment where adverse events can have serious consequences. Drawing from this analogy, the concept of surgical data recorders (SDRs) has emerged to bring similar data-driven accountability and insight into surgical practice [3].

SDRs are integrated systems that collect multimodal intraoperative data, including video and audio recordings, patient physiological signals, and device metrics. Unlike traditional reviews of adverse surgical outcomes, which often rely on limited documentation from anesthesia protocols, surgery reports, or subjective recollection, SDRs enable comprehensive, continuous, and objective data capture. This approach minimizes common limitations such as recall bias, the observer effect, and inconsistent reporting [4-9].

Early efforts in intraoperative performance assessment focused largely on single-channel video feeds of laparoscopic equipment [5]. While video remains a core element, recent innovations have expanded SDRs to include audio, kinematic data, and even eye-tracking technologies, offering a more complete picture of team dynamics and procedural flow. Among the leading systems currently in clinical use are the OR Black Box (Surgical Safety Technologies Inc) and the caresyntax solution (Caresyntax), which have been implemented across multiple continents and institutions [3,10,11].

The timing of this review is critical. In recent years, SDRs have moved beyond pilot implementations and are increasingly integrated into hospital infrastructures. This expansion, coupled with rapid advancements in artificial intelligence and analytics, is positioning SDRs to evolve from passive observational tools into platforms capable of real-time feedback, predictive modeling, and decision support [11]. As these technologies mature, understanding their current clinical applications becomes essential [12].

Despite growing interest and increasing publications on SDRs, the literature remains fragmented. The purposes of SDR deployment are diverse, ranging from surgical performance assessment to workflow analysis and quality improvement. However, there is no comprehensive synthesis of how these systems are being applied, what types of clinical data are being collected, and which outcomes are being influenced. A clear categorization of SDR use cases, research domains, and methodological trends is needed to understand their broader potential, guide best practices, and accelerate their integration into routine surgical care [4].

Therefore, the aim of this systematic review is to critically examine and synthesize the existing literature on the clinical application of SDRs, with a focus on identifying the types of studies conducted, the primary areas of investigation, and the methodological quality of the evidence. By mapping these domains, this review seeks to clarify how SDRs are currently being used, where their greatest potential lies, and what gaps remain for future research.

## Methods

### Registration and Protocol

The protocol of this study was prospectively registered (PROSPERO CRD42024527164) on April 1, 2024. At the time of preregistration, the literature search and selection process were piloted. The protocol was developed according to the Preferred Reporting Items for Systematic Reviews and Meta-Analysis (PRISMA) [13].

### Search Strategy

The literature was systematically reviewed by conducting a comprehensive electronic search of the MEDLINE (PubMed), Excerpta medica database (EMBASE) and Web of Science databases. References of the retrieved studies were manually searched to identify more potentially relevant studies. The search was rerun on February 10, 2025. The following search terms were used: “operating room black box,” “caresyntax,” “surgical data recording,” and “OR Black Box.” These terms were deliberately selected to focus the search on systems capable of multisource intraoperative data capture, in line with the review’s inclusion criteria, and to minimize irrelevant results from single-channel recording systems. No filters or restrictions were applied. Articles in all languages were considered.

### Study Selection

Results were screened independently by 2 authors (NS and AR) based on title and abstract for relevance and assessment for inclusion based on full text. Articles were evaluated via Covidence, a software platform that allows blinded assessment of articles based on title, abstract, and uploaded full text by multiple reviewers [14]. If there was disagreement on articles screened for eligibility based on title and abstract, they were automatically forwarded to full-text screening. Conflicts over the inclusion of articles for assessment following full-text screening were resolved by consulting a third reviewer (TP).

### Eligibility Criteria

To be included, studies needed to evaluate SDR systems capable of multichannel data capture—that is, devices recording and integrating data from multiple sources such as video, audio, physiological monitoring, and surgical instruments. This distinction was essential to differentiate comprehensive SDR platforms from studies using single-modality systems, such as isolated camera recordings manually analyzed post hoc, which lack real-time integration and broader environmental context. There were no language restrictions. Literature focusing on the implementation or legal evaluation of SDRs was excluded. Literature for which full text was not available was also excluded.

### Risk of Bias Assessment

A modified version of the Risk Of Bias In Non-randomized Studies - of Exposure (ROBINS-E) risk-assessment tool from the Cochrane Method group was used [15]. As most studies were observational, cross-sectional, or cohort studies without a specific exposure or intervention, we focused the quality assessment on 5 domains: confounding, bias in selection of participants, bias due to missing data, bias in measurement of the outcome, and bias in selection of the reported result. A study was considered to have high overall bias if 2 of the 5 domains were considered to be high risk. A total of 2 reviewers (NS and AR) independently assessed the risk of bias. Any disagreement was resolved by consultation with a third reviewer (TP).

### Data Extraction and Statistics

Categorical, metadata, and numerical data were extracted manually by 2 reviewers (NS and AR) using data extraction forms. Data extraction was verified by 2 additional reviewers (GP and GS). The included papers were searched for quantifiable data that were assessed in more than 2 studies. If the quantifiable data was not provided in rates stating the events observed per minute, they were calculated using the data provided. This was done, where necessary, by converting events per case to events per minute when case duration was reported. Statistical analysis was conducted using R (version 4.3.1; R Foundation for Statistical Computing) [16], *metafor* [17] and the *ggplot2* package [18]. Heterogeneity among studies was assessed using *I*² statistics. A random-effects meta-analysis was conducted to account for the heterogeneity among studies. The restricted maximum likelihood estimator was used to estimate between-study variance (τ²), as it provides an unbiased and efficient estimate of heterogeneity, particularly for small sample sizes. The effect sizes (eg, means or standardized mean differences) were weighted using inverse-variance weighting. A forest plot was generated to visualize individual study estimates and the pooled effect size.

## Results

### Study Characteristics

**Figure 1.**
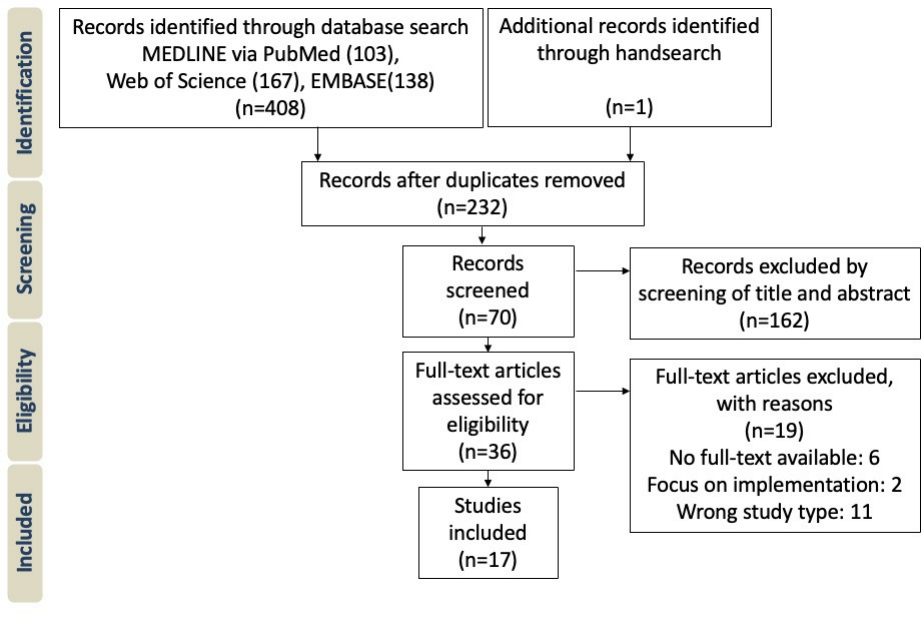
Preferred Reporting Items for Systematic Reviews and Meta-Analyses (PRISMA) flow chart (study identification and screening process).

Figure 1 shows a detailed overview of the selection process. A total of 408 records were retrieved, 103 from MEDLINE via PubMed, 138 from Embase, and 167 from Web of Science. An additional record was identified through hand search. After removing duplicates, 70 records were screened based on title and abstract, resulting in 36 records being evaluated for eligibility based on full-text screening. A total of 6 studies were excluded due to lack of full text and 11 due to incorrect study type, meaning that the devices were not capable of recording data from multiple sources or were not used in a clinical setting. In addition, 2 studies solely examined implementation of surgical recording devices and were excluded as well. A total of 17 studies remained to be included in qualitative and quantitative analysis. The 17 studies included in this systematic review were published from 2018 to 2024. All 17 studies included used the OR Black Box. It was noted that the studies almost exclusively originated in the United States and Canada and were mostly conducted in a general surgery setting (see Table 1).

**Table 1.** Summary of the 17 included studies.

Author, year	Country	Type of study	Patients, n	Specialty	Category
Al Abbas et al, 2022 [8][Bibr R8]	The United States	Interventional study	3879	Multiple	Safety
Ayas et al, 2022 [19][Bibr R19]	Canada	Mixed-methods study	40	General surgery	Safety
Doyen et al, 2020 [20][Bibr R20]	Belgium	Observational study	1	Vascular surgery	Technical skills assessment and safety
Doyen et al, 2023 [21][Bibr R21]	Belgium	Cohort study	22	Vascular surgery	Technical skills assessment
Etherington et al, 2023 [22][Bibr R22]	Canada	Descriptive study	25	Gynecology	Behavior in the OR^a^
Fecso et al. 2018 [23][Bibr R23]	Canada	Observational study	56	General surgery	Technical skills assessment
Gabrielli et al, 2020 [24][Bibr R24]	Canada	Cohort study	50	General surgery	Economic and safety
Grantcharov et al, 2019 [25][Bibr R25]	The United States	Observational study	23	General surgery	Behavior in the OR
Incze et al, 2024 [26][Bibr R26]	Canada	Observational study	23	General surgery	Behavior in the OR
Jung et al, 2019 [27][Bibr R27]	Canada	Observational study	131	General surgery	Safety
Jung et al, 2020 [10][Bibr R10]	Canada	Cohort study	132	General surgery	Safety
Kumar et al, 2023 [11][Bibr R11]	India	Cross-sectional study	52	General surgery	Economic
Nensi et al, 2021 [4][Bibr R4]	Canada	Cross-sectional study	25	Gynecology	Economic and technical skills assessment
Rai et al, 2021 [28][Bibr R28]	The United States	Cohort study	80	Urology	Safety
Riley et al, 2024 [29][Bibr R29]	The United States and Canada	Cohort study	7127	N/A^b^	Safety
Soensen et al, 2023 [30][Bibr R30]	Belgium	Cohort study	22	Vascular surgery	Behavior in the OR
Al Abbas et al, 2024 [31][Bibr R31]	The United States	Cohort study	4581	Multiple	Safety

a OR: operating room.

b N/A: not available.

All included papers were classified into categories that reflected their principal topics. The main categories in which the analyzed studies fall into are economic, safety, behavior in the OR, and technical skills assessment. Articles covered between 1 and 3 categories.

### Quality Assessment: Risk of Bias

A total of 9 of the 17 studies were found to have a low overall bias [8,10,11,19,22,26,27,29-31]. However, bias due to confounding was a significant issue. Largely due to the small sample sizes and a relatively short study period, the Hawthorne effect on the study population could not be excluded. In addition, selection bias was evident, with the authors preferring to demonstrate the black box capabilities using routine cases. Furthermore, the study populations were reduced due to dropout, either due to patients or staff not consenting to participate. Due to prospectively collecting the data in the OR, the risk for reporting bias is low (see Table 2). It was found that 13 of the 17 studies had the founder of the OR Black Box as a co-author (see Multimedia Appendix 1), which is another possible source of bias.

**Table 2.** Modified Risk Of Bias In Non-Randomized Studies - of Exposure (ROBINS-E) bias assessment.

Authors	D1^a^	D2^b^	D3^c^	D4^d^	D5^e^
Al Abbas et al 2022 [8][Bibr R8]	Low bias	Low bias	Low bias	Low bias	Low bias
Ayas et al 2022 [19][Bibr R19]	Low bias	Low bias	High bias	Low bias	Low bias
Doyen et al 2020 [20][Bibr R20]	High bias	High bias	Low bias	Low bias	High bias
Doyen et al 2023 [21][Bibr R21]	High bias	High bias	Low bias	Low bias	Low bias
Etherington et al 2023 [22][Bibr R22]	Low bias	Low bias	Low bias	Low bias	Low bias
Fecso et al 2018 [23][Bibr R23]	High bias	High bias	High bias	Low bias	Low bias
Gabrielli et al 2020 [24][Bibr R24]	High bias	High bias	Low bias	Low bias	Low bias
Grantcharov et al 2019 [25][Bibr R25]	High bias	High bias	Low bias	Low bias	Low bias
Incze et al 2024 [26][Bibr R26]	High bias	Low bias	Low bias	Low bias	Low bias
Jung et al 2019 [27][Bibr R27]	High bias	Low bias	Low bias	Low bias	Low bias
Jung et al 2020 [10][Bibr R10]	High bias	Low bias	Low bias	Low bias	Low bias
Kumar et al 2023 [11][Bibr R11]	Low bias	Low bias	High bias	Low bias	Low bias
Nensi et al 2021 [4][Bibr R4]	High bias	High bias	Low bias	Low bias	Low bias
Rai et al 2021 [28][Bibr R28]	Low bias	High bias	Low bias	Low bias	High bias
Riley et al 2024 [29][Bibr R29]	Low bias	Low bias	Low bias	Low bias	Low bias
Soensen et al 2023 [30][Bibr R30]	Low bias	High bias	Low bias	Low bias	Low bias
Al Abbas et al 2024 [31][Bibr R31]	Low bias	Low bias	Low bias	Low bias	Low bias

a D1: Risk of bias due to confounding.

b D2: Risk of bias in selection of participants into the study.

c D3: Risk of bias due to missing data.

d D4: Risk of bias arising from measurement of the outcome.

e D5: Risk of bias in selection of the reported result.

### Economic Use

**Figure 2.**
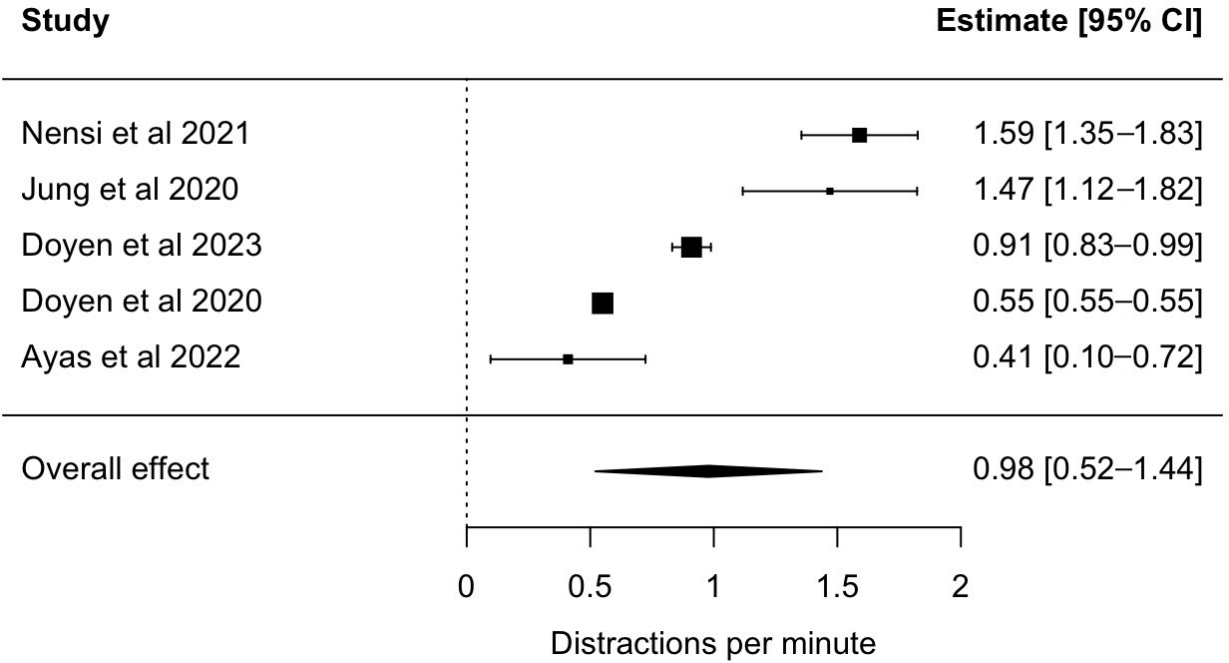
Distractions per minute. Values show scatter. Overall, about one distraction per minute was noted [4,10,19,20,21].

A total of 3 studies shed light on the economic use of SDRs, with a particular focus on the analysis of operating time and the factors that contribute to it. A cohort study conducted at a tertiary care teaching hospital in Canada used a SDR to assess the effect of 3-dimensional versus 2-dimensional imaging for 50 elective laparoscopic Roux-en-Y Gastric Bypass surgery cases on adults on duration of surgery and showed that the use of 3-dimensional imaging systems reduces operating time [24]. A second cross-sectional study at this hospital evaluated duration of procedures and their procedural steps in a cohort of 25 patients undergoing elective total laparoscopic hysterectomy. The authors concluded that assessing the median duration of each step allows for benchmarking and seeking areas of improvement [4]. However, it has not been assessed whether benchmarking improved operating time. Intraoperative distractions, namely time pressure, opening of doors, and machine alarms were noted as well [4]. By observing 51 cases in a cross-sectional study in a general surgery department in a tertiary care hospital in India, the types of distraction, being related to other staff or technical issues, which occur during surgery could be identified. It was shown that the number of distractions correlated with the length of total operating time [11]. Although not shown in these studies in relation to SDRs, prolonging operating time causes not only cost but also has a negative effect on patient safety [32]. The number of distractions per minute was a variable that was frequently assessed. The results demonstrated that the papers collectively reported around one distraction per minute (mean 0.98, 95% CI 0.52-1.44; see Figure 2). *I*² statistical evaluation (*I*²=97.7%) showed high heterogeneity in the results.

### Safety

**Figure 3.**
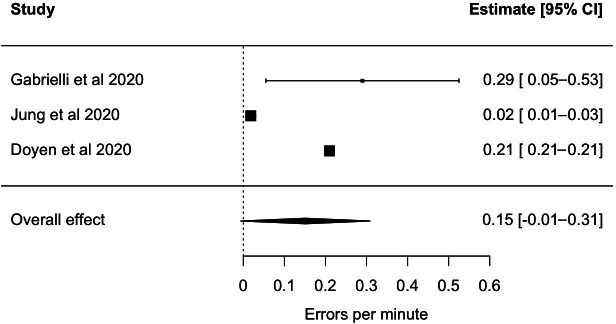
Errors per minute. Values show scatter. Overall, about 1 distraction per 6 minutes was noted [10,20,24].

The effect of SDR use regarding OR safety was demonstrated in 8 studies [8,10,19,20,24,27-29]. These studies have focused on 2 key areas: first, the impact of SDRs on patient safety, analyzing errors, adverse events, and adherence to checklists [8,10,19,24,27-29]; and second, the impact of SDRs on personnel safety in the context of radiation protection [20]. The study by Gabrielli et al [24] demonstrated the superiority of 3D imaging compared to 2D imaging and therefore its contribution to a safer OR. Another study of 131 elective laparoscopic cases at a tertiary care hospital in Canada compared adverse event identification with an SDR to documentation review regarding Verres Needle Injuries. Verres needles are blindly placed in laparoscopic surgery to create the pneumoperitoneum. Injuries to the underlying tissue and organs tend to happen and are a feared complication [33]. Using a SDR especially allowed for assessment of near misses regarding possible injuries. Although these near misses did not cause further adverse events, the authors argued that they should be seen as small errors that can contribute to adverse events, as illustrated by the “Swiss cheese model” [27]. This may prompt a more accurate review on morbidity and mortality rounds since errors without them leading to adverse events can be discussed [10]. The incidence of adverse events in urological settings was also examined in 80 cases at a tertiary care hospital in the United States. The findings indicated that such events occur with high frequency during all phases of surgical procedures [28]. Overall, 3 studies shown in Figure 3 quantified the occurrence rate of errors (mean 0.15, 95% CI −0.01 to 0.31; see Figure 3). *I*² statistical evaluation (*I*²=89.8%) showed high heterogeneity in the results. Other studies focused on personnel safety. In an Angio-Suite, the safety behavior during an endovascular aortic repair was assessed. Postoperative assessment showed areas of improvement regarding the use of radiation exposing reducing techniques. Regarding the use of the World Health Organization (WHO) Surgical Safety Checklist compliance, engagement, and quality were monitored first in a tertiary care hospital in the United States. In this interventional study, 3879 laparoscopic procedures were used to acknowledge weaknesses of the debrief process [8]. Second, the compliance, engagement, and quality could be monitored in seven different North American academic medical centers compiling 7243 surgical procedures. Here the authors found that the introduction of team members led to a higher engagement in the time-out, which underlines the importance of proper team introduction. Furthermore, in line with previous literature, poorer performance in the debrief was found [29]. Corroborating the implementation study of the WHO Surgical Safety Checklist [34], Al Abbas et al [31] showed that for the use of an SDR monitoring checklist compliance in 4581 cases across multiple surgical specialties in a tertiary care hospital in the United States, an observed greater adherence to the checklist results resulted in improved postoperative patient outcomes.

### Behavior in the OR

Broadening the insight of the cause of safety-restricting situations, four studies investigated the OR from an organizational psychology perspective [22,25,26,30]. Understanding the roles of the interprofessional team members was investigated in a large community teaching hospital on 23 general surgery laparoscopic cases. They observed that during intraoperative adverse events, nurses expressed more backup behavior, whereas surgeons provided psychological support for each other and took leadership [26]. Another study of elective routine endovascular procedures at a tertiary care hospital in Belgium showed how leadership style fluctuated by operative phase and that the presence of a transformational leadership style improves team behavior, meaning that team members are more open, share more knowledge, and collaborate better [30]. Measuring heart rate variability in an attending surgeon via electrocardiogram as a surrogate for stress, linking mental stress to worse technical surgical performance, was done in a different study in 25 general surgery cases at a tertiary care hospital in the United States of America [25]. By focusing on the anesthesiologic team and their involvement in 25 gynecological procedures, a study conducted at a tertiary care hospital in Canada revealed that anesthesiologists demonstrated exemplary performance in situational awareness and teamwork [22].

### Technical Skills Assessment

By reviewing intraoperative data, these studies allowed for technical skills assessment and for revelation of the underlying mechanisms that lead to adverse events, errors, and threats. For example, a study of Nensi et al [4] highlights the importance of adequate cauterization of uterine arteries to prevent unexpected bleeding. Other research described surgical errors, events, and distractions that occurred during 132 laparoscopic general surgery procedures at a tertiary care hospital in Canada. In this study, attending surgeons were shown to be most technically proficient compared to fellows and residents [10]. In a cohort study at a tertiary care center in Belgium in an Angio-Suite with 22 endovascular cases, low technical skills were associated with intraoperative complications [21]. One study examined the relationship between technical and nontechnical behavior. It was suggested that negative nontechnical behavior was associated with errors. In addition, common errors and their causes, such as inadequate visualization and grasping force during 56 laparoscopic Roux-en-Y gastric bypass cases at a Canadian tertiary care center, were identified, allowing for the identification of areas for improvement [23].

## Discussion

### Principal Findings

This systematic review synthesizes existing literature on SDRs and the OR Black Box as the representative system of integrated data capture technologies inspired by the aviation industry. While there are observational studies that use standalone camera setups to retrospectively analyze intraoperative behavior or events [9], these typically do not qualify as SDRs under our definition, as they lack the integration of multiple synchronous data streams. SDRs, in contrast, offer a continuous, systematized capture of various intraoperative parameters, enabling automated or semiautomated analysis and contextual insights that go beyond what can be gleaned from video alone. These devices optimize OR efficiency and ultimately enhance patient safety by providing insights into the OR that were previously not possible to obtain. This is achieved by enabling the understanding of both technical and nontechnical variations, adverse events, and the interaction between team members.

The articles included demonstrate that the installed SDRs facilitate the precise documentation of case times and procedural steps. Furthermore, the occurrence of distractions per minute could be documented. As a consequence of the heterogeneity of the definition of distractions, a wide range of distraction rates was observed, which suggests that most of the variation between studies is due to real differences in study populations, methodologies, or effects, rather than random chance. However, the observed distractions (mean 98/min, 95% CI 0.52-1.44) align with the rates documented in the literature based on direct observation, which range from 0.29 to 1.2/min [35-38]. Nevertheless, it is important to note that the values exhibit considerable scatter due to definitional, demographic, and methodological differences across studies. A study by Nensi et al [4] demonstrated that distractions can also contribute to delays. Furthermore, another study suggested that distractions may have positive aspects, such as the opportunity to share patient information or reducing stress in the team, which can ultimately contribute to patient safety [19].

From an economic standpoint, it would be interesting to determine whether the use of SDRs can enhance operational efficiency through benchmarking and evaluation, with the objective of optimizing workflows. With regard to safety, it was possible to assess the rate of adverse events and categorize them. Although errors were evaluated through direct observation prior to the implementation of SDRs, the precise numerical data and occurrence rate were vaguely reported. SDRs could be used to quantify the incidence of errors, but there is heterogeneity due to definitional, demographic, and methodological differences between studies. In the reported studies, a preliminary attempt was made to link the incidence of adverse events to other assessed metrics, such as nontechnical skills, in order to gain insight into their underlying causes. To permit an assessment of the significance of these connections, the studies were likely underpowered with respect to the number of observed cases. Further it would be interesting to consider whether the assessment of critical events in real time could also provide feedback in real time, as is already done in some cases [20,39,40]. By applying an organizational psychology perspective, the present studies demonstrated the impact of different behaviors on the team dynamic and well-functioning teams identified [22]. This knowledge can be advanced, and other OR teams can benefit from this by applying a transformational leadership style [30]. As it has already been demonstrated using straightforward video recordings, SDRs provide a multisource approach, which was underused in regard to patient physiological data, that enables the assessment of technical skills and issues across a range of training levels and facilitates training [10,24]. The next step is to demonstrate how this can be effectively implemented in practice. As application of SDRs in surgery and scientific evaluation of SDR is an emerging field, most of the studies were in an early phase of evidence generation and focused on demonstrating capabilities, such as capturing distractions, operating time, and technical and nontechnical behavior, in a descriptive way. Observing how adherence to surgical checklists changed due to an intervention and looked promising, and analyzing how other interventions are reflected in the OR, was already suggested [26,30]. The SDRs were used in a general, vascular, urology, and gynecology setting. It would be insightful to establish these recorders and conduct research in other ORs. The integration of other sensors, as shown in a study measuring the surgeon’s heart rate variability, may provide further insight [25]. Perhaps sensors in laparoscopic surgical instruments could record after what length of time or what actions they fail.

SDRs are essential for linking intraoperative factors with outcomes, but this raises privacy concerns as it may breach the platform’s anonymity. To ensure high inclusion rates and gain ethical committee approval for future studies, it is crucial to find solutions to these privacy issues [41]. One approach could be an honest broker system that ensures the privacy of patient data while providing deidentified patient data to researchers [31,42].

### Limitations

While our search terms were designed to capture a broad range of SDR technologies, only studies involving the OR Black Box met the inclusion criteria for this review. This reflects the current publication landscape rather than an intentional exclusion, but nonetheless limits the scope of our analysis to a single SDR. It is important to consider that the studies were testing the OR Black Box in a preliminary state and did not compare it to other methods of data gathering in the OR when interpreting the results of this systematic review. For this reason, only a single-arm meta-analysis was conducted. As a significant number of the studies are partly authored by the founder of the OR Black Box, there is a possibility of more favorable reporting of its capabilities [43]. Future research by independent teams and across diverse SDR platforms will be crucial to validate these early findings and mitigate the influence of commercial or authorship bias.

### Conclusions

As a representative system, the SDRs and OR Black Box, provide an unobstructed view into the OR, potentially improving the OR economically, improving safety, showing how the OR behaves, and allowing technical skills assessment. With an advanced toolbox of surgical recording devices using artificial intelligence, more areas of study will be developed, and broader use and less time-consuming analysis will be possible [20,21]. Finally, SDRs offer valuable insights that minimize the Hawthorne effect after an implementation period [44] and vast opportunities for further research lie ahead.

## Supplementary material

10.2196/72703 Multimedia Appendix 1 Articles co-authored by the founder of the OR Black Box.

10.2196/72703 Checklist 1 PRISMA (Preferred Reporting Items for Systematic Reviews and Meta-Analyses) 2020 checklist.

## Abbreviations

OR: operating room
PRISMA: Preferred Reporting Items for Systematic Reviews and Meta-Analyses
ROBINS-E: Risk Of Bias In Non-randomized Studies - of Exposure
SDR: surgical data recorder
WHO: World Health Organization

## References

[1] Vidović A, Franjić A, Štimac I, Ban MO (2022). The importance of flight recorders in the aircraft accident investigation. Transportation Research Procedia.

[2] Kontogiannis T, Malakis S (2009). A proactive approach to human error detection and identification in aviation and air traffic control. Saf Sci.

[3] Goldenberg MG, Jung J, Grantcharov TP (2017). Using data to enhance performance and improve quality and safety in surgery. JAMA Surg.

[R4] Nensi A, Palter V, Reed C (2021). Utilizing the operating room black box to characterize intraoperative delays, distractions, and threats in the gynecology operating room: a pilot study. Cureus.

[5] Bowermaster R, Miller M, Ashcraft T (2015). Application of the aviation black box principle in pediatric cardiac surgery: tracking all failures in the pediatric cardiac operating room. J Am Coll Surg.

[6] Shah NA, Jue J, Mackey TK (2020). Surgical data recording technology: a solution to address medical errors?. Ann Surg.

[7] Kompier MA (2006). The “Hawthorne effect” is a myth, but what keeps the story going?. Scand J Work Environ Health.

[R8] Al Abbas AI, Sankaranarayanan G, Polanco PM (2022). The operating room black box: understanding adherence to surgical checklists. Ann Surg.

[9] Levin M, McKechnie T, Kruse CC, Aldrich K, Grantcharov TP, Langerman A (2021). Surgical data recording in the operating room: a systematic review of modalities and metrics. Br J Surg.

[R10] Jung JJ, Jüni P, Lebovic G, Grantcharov T (2020). First-year analysis of the operating room black box study. Ann Surg.

[R11] Kumar A P, Pratik PP, Ravichandran N (2023). Operating room black box: scrutinizer of theatre practices. Laparoscopic, Endoscopic and Robotic Surgery.

[12] Maier-Hein L, Eisenmann M, Sarikaya D (2022). Surgical data science - from concepts toward clinical translation. Med Image Anal.

[13] Page MJ, McKenzie JE, Bossuyt PM (2021). The PRISMA 2020 statement: an updated guideline for reporting systematic reviews. BMJ.

[14] Systematic review tool. Covidence.

[15] Higgins JPT, Morgan RL, Rooney AA (2024). A tool to assess risk of bias in non-randomized follow-up studies of exposure effects (ROBINS-E). Environ Int.

[16] (2021). R: a language and environment for statistical computing. R Project.

[17] Viechtbauer W (2010). Conducting meta-analyses in R with the metafor package. J Stat Softw.

[18] Valero-Mora PM (2010). ggplot2: elegant graphics for data analysis. Journal of Statistical Software, Book Reviews.

[R19] Ayas S, Donmez B, Kazlovich K, Lombardi S, Jain A (2022). The prevalence and potential effects of distractions in general surgery: a mixed-methods study. Proceedings of the Human Factors and Ergonomics Society Annual Meeting.

[R20] Doyen B, Gordon L, Soenens G (2020). Introduction of a surgical black box system in a hybrid angiosuite: challenges and opportunities. Phys Med.

[R21] Doyen B, Soenens G, Maurel B (2023). Assessing endovascular team performances in a hybrid room using the Black Box system: a prospective cohort study. J Cardiovasc Surg (Torino).

[R22] Etherington C, Burns JK, Ghanmi N (2023). Identifying positive and negative use of non-technical skills by anesthesiologists in the clinical operating room: an exploratory descriptive study. Heliyon.

[R23] Fecso AB, Kuzulugil SS, Babaoglu C, Bener AB, Grantcharov TP (2018). Relationship between intraoperative non-technical performance and technical events in bariatric surgery. Br J Surg.

[R24] Gabrielli ME, Saun TJ, Jung JJ, Grantcharov TP (2020). Assessment of 3-dimensional vs 2-dimensional imaging and technical performance using a multiport intraoperative data capture and analytic system for patients undergoing laparoscopic roux-en-y gastric bypass surgery. JAMA Netw Open.

[R25] Grantcharov PD, Boillat T, Elkabany S, Wac K, Rivas H (2019). Acute mental stress and surgical performance. BJS Open.

[R26] Incze T, Pinkney SJ, Li C (2024). Using the operating room black box to assess surgical team member adaptation under uncertainty: an observational study. Ann Surg.

[R27] Jung JJ, Adams-McGavin RC, Grantcharov TP (2019). Underreporting of Veress needle injuries: comparing direct observation and chart review methods. J Surg Res.

[R28] Rai A, Beland L, Aro T, Jarrett M, Kavoussi L (2021). Patient safety in the operating room during urologic surgery: The OR Black Box experience. World J Surg.

[R29] Riley MS, Etheridge J, Palter V (2024). Remote assessment of real-world surgical safety checklist performance using the OR Black Box: a multi-institutional evaluation. J Am Coll Surg.

[R30] Soenens G, Marchand B, Doyen B, Grantcharov T, Van Herzeele I, Vlerick P (2023). Surgeons’ leadership style and team behavior in the hybrid operating room: prospective cohort study. Ann Surg.

[R31] Al Abbas AI, Meier J, Daniel W (2024). Impact of team performance on the surgical safety checklist on patient outcomes: an operating room black box analysis. Surg Endosc.

[32] Subhas G, Gupta A, Bhullar J (2011). Prolonged (longer than 3 hours) laparoscopic cholecystectomy: reasons and results. Am Surg.

[33] Azevedo JLMC, Azevedo OC, Miyahira SA (2009). Injuries caused by Veress needle insertion for creation of pneumoperitoneum: a systematic literature review. Surg Endosc.

[34] de Jager E, Gunnarsson R, Ho YH (2019). Implementation of the world health organization surgical safety checklist correlates with reduced surgical mortality and length of hospital admission in a high-income country. World J Surg.

[35] van Harten A, Gooszen HG, Koksma JJ, Niessen TJH, Abma TA (2021). An observational study of distractions in the operating theatre. Anaesthesia.

[36] Persoon MC, Broos HJHP, Witjes JA, Hendrikx AJM, Scherpbier AJJM (2011). The effect of distractions in the operating room during endourological procedures. Surg Endosc.

[37] Pereira BMT, Pereira AMT, Correia CDS, Marttos AC, Fiorelli RKA, Fraga GP (2011). Interruptions and distractions in the trauma operating room: understanding the threat of human error. Rev Col Bras Cir.

[38] Healey AN, Sevdalis N, Vincent CA (2006). Measuring intra-operative interference from distraction and interruption observed in the operating theatre. Ergonomics.

[39] Schulthess P, Bohnen J, Grantcharov T, Palter V (2020). The or black box nursing education curriculum: using video review to optimize patient safety. AORN J.

[40] van Dalen ASHM, Jansen M, van Haperen M (2021). Implementing structured team debriefing using a Black Box in the operating room: surveying team satisfaction. Surg Endosc.

[41] van Dalen ASHM, Legemaate J, Schlack WS, Legemate DA, Schijven MP (2019). Legal perspectives on black box recording devices in the operating environment. Br J Surg.

[42] Dhir R, Patel AA, Winters S (2008). A multidisciplinary approach to honest broker services for tissue banks and clinical data: a pragmatic and practical model. Cancer.

[43] Probst P, Knebel P, Grummich K (2016). Industry bias in randomized controlled trials in general and abdominal surgery: an empirical study. Ann Surg.

[44] McCambridge J, Witton J, Elbourne DR (2014). Systematic review of the Hawthorne effect: new concepts are needed to study research participation effects. J Clin Epidemiol.

