# Supplementary material for: Enhancing Surgical Safety and Efficiency: Systematic Review and Single-Arm Meta-Analysis of Surgical Data Recorders
Source: J Med Internet Res. 2025 Jul 28;27:e72703. doi: 10.2196/72703 (PMC12303543; doi:10.2196/72703)
Supplement: Multimedia Appendix 1 [file jmir-v27-e72703-s001.docx]

Table S1. Articles co-authored by the founder of the OR Black Box.

| Study | Co-authored |
| --- | --- |
| Al Abbas et al. 2022 [1] | Yes |
| Ayas et al. 2022 [2] | No |
| Doyen et al. 2020 [3] | Yes |
| Doyen et al. 2023 [4] | Yes |
| Etherington et al. 2023 [5] | No |
| Fecso et al. 2018 [6] | Yes |
| Gabrielli et al. 2020 [7] | Yes |
| Grantcharov et al. 2019 [8] | Yes |
| Incze et al. 2024 [9] | Yes |
| Jung et al. 2019 [10] | Yes |
| Jung et al. 2020 [11] | Yes |
| Kumar et al. 2023 [12] | No |
| Nensi et al. 2021 [13] | Yes |
| Rai et al. 2021 [14] | No |
| Riley et al. 2024 [15] | Yes |
| Soensen et al. 2023 [16] | Yes |
| Al Abbas et al. 2024 [17] | Yes |

References

1. Al Abbas AI, Sankaranarayanan G, Polanco PM, et al. The operating room black box: understanding adherence to surgical checklists. Ann Surg. Dec 1, 2022;276(6):995-1001. [doi: 10.1097/SLA.0000000000005695] [Medline: 36120866]
2. Ayas S, Donmez B, Kazlovich K, Lombardi S, Jain A. The prevalence and potential effects of distractions in general surgery: a mixed-methods study. Proceedings of the Human Factors and Ergonomics Society Annual Meeting. Sep 2022;66(1):2132-2136. [doi: 10.1177/1071181322661335]
3. Doyen B, Gordon L, Soenens G, et al. Introduction of a surgical black box system in a hybrid angiosuite: challenges and opportunities. Phys Med. Aug 2020;76:77-84. [doi: 10.1016/j.ejmp.2020.06.013] [Medline: 32615511]
4. Doyen B, Soenens G, Maurel B, et al. Assessing endovascular team performances in a hybrid room using the Black Box system: a prospective cohort study. J Cardiovasc Surg (Torino). Feb 2023;64(1):82-92. [doi: 10.23736/S0021-9509.22.12226-3] [Medline: 36168949]
5. Etherington C, Burns JK, Ghanmi N, et al. Identifying positive and negative use of non-technical skills by anesthesiologists in the clinical operating room: an exploratory descriptive study. Heliyon. Mar 2023;9(3):e14094. [doi: 10.1016/j.heliyon.2023.e14094] [Medline: 36938432]
6. Fecso AB, Kuzulugil SS, Babaoglu C, Bener AB, Grantcharov TP. Relationship between intraoperative non-technical performance and technical events in bariatric surgery. Br J Surg. Jul 2018;105(8):1044-1050. [doi: 10.1002/bjs.10811] [Medline: 29601079]
7. Gabrielli ME, Saun TJ, Jung JJ, Grantcharov TP. Assessment of 3-dimensional vs 2-dimensional imaging and technical performance using a multiport intraoperative data capture and analytic system for patients undergoing laparoscopic roux-en-y gastric bypass surgery. JAMA Netw Open. Jan 3, 2020;3(1):e1920084. [doi: 10.1001/jamanetworkopen.2019.20084] [Medline: 31995217]
8. Grantcharov PD, Boillat T, Elkabany S, Wac K, Rivas H. Acute mental stress and surgical performance. BJS Open. Feb 2019;3(1):119-125. [doi: 10.1002/bjs5.104] [Medline: 30734023]
9. Incze T, Pinkney SJ, Li C, et al. Using the operating room black box to assess surgical team member adaptation under uncertainty: an observational study. Ann Surg. Jul 1, 2024;280(1):75-81. [doi: 10.1097/SLA.0000000000006191] [Medline: 38193296]
10. Jung JJ, Adams-McGavin RC, Grantcharov TP. Underreporting of Veress needle injuries: comparing direct observation and chart review methods. J Surg Res. Apr 2019;236(266-70):266-270. [doi: 10.1016/j.jss.2018.11.039] [Medline: 30694765]
11. Jung JJ, Jüni P, Lebovic G, Grantcharov T. First-year analysis of the operating room black box study. Ann Surg. Jan 2020;271(1):122-127. [doi: 10.1097/SLA.0000000000002863] [Medline: 29916872]
12. Kumar A P, Pratik PP, Ravichandran N. Operating room black box: scrutinizer of theatre practices. Laparoscopic, Endoscopic and Robotic Surgery. Dec 2023;6(4):142-146. [doi: 10.1016/j.lers.2023.10.002]
13. Nensi A, Palter V, Reed C, et al. Utilizing the operating room black box to characterize intraoperative delays, distractions, and threats in the gynecology operating room: a pilot study. Cureus. Jul 2021;13(7):e16218. [doi: 10.7759/cureus.16218] [Medline: 34367818]
14. Rai A, Beland L, Aro T, Jarrett M, Kavoussi L. Patient safety in the operating room during urologic surgery: The OR Black Box experience. World J Surg. Nov 2021;45(11):3306-3312. [doi: 10.1007/s00268-021-06251-9] [Medline: 34351487]
15. Riley MS, Etheridge J, Palter V, et al. Remote assessment of real-world surgical safety checklist performance using the OR Black Box: a multi-institutional evaluation. J Am Coll Surg. Feb 1, 2024;238(2):206-215. [doi: 10.1097/XCS.0000000000000893] [Medline: 37846086]
16. Soenens G, Marchand B, Doyen B, Grantcharov T, Van Herzeele I, Vlerick P. Surgeons’ leadership style and team behavior in the hybrid operating room: prospective cohort study. Ann Surg. Jul 1, 2023;278(1):e5-e12. [doi: 10.1097/SLA.0000000000005645] [Medline: 35904023]
17. Al Abbas AI, Meier J, Daniel W, et al. Impact of team performance on the surgical safety checklist on patient outcomes: an operating room black box analysis. Surg Endosc. Oct 2024;38(10):5613-5622. [doi: 10.1007/s00464-024-11064-7] [Medline: 39103662]
